# Differential IL-1β secretion by monocyte subsets is regulated by Hsp27 through modulating mRNA stability

**DOI:** 10.1038/srep39035

**Published:** 2016-12-15

**Authors:** Eva Hadadi, Biyan Zhang, Kajus Baidžajevas, Nurhashikin Yusof, Kia Joo Puan, Siew Min Ong, Wei Hseun Yeap, Olaf Rotzschke, Endre Kiss-Toth, Heather Wilson, Siew Cheng Wong

**Affiliations:** 1 University of Sheffield, Dept of Infection, Immunity & Cardiovascular Disease (IICD), Sheffield, UK; 2 Singapore Immunology Network (SIgN), Agency for Science, Technology and Research (ASTAR), Singapore

## Abstract

Monocytes play a central role in regulating inflammation in response to infection or injury, and during auto-inflammatory diseases. Human blood contains classical, intermediate and non-classical monocyte subsets that each express characteristic patterns of cell surface CD16 and CD14; each subset also has specific functional properties, but the mechanisms underlying many of their distinctive features are undefined. Of particular interest is how monocyte subsets regulate secretion of the apical pro-inflammatory cytokine IL-1β, which is central to the initiation of immune responses but is also implicated in the pathology of various auto-immune/auto-inflammatory conditions. Here we show that primary human non-classical monocytes, exposed to LPS or LPS + BzATP (3’-*O*-(4-benzoyl)benzyl-ATP, a P2X7R agonist), produce approx. 80% less IL-1β than intermediate or classical monocytes. Despite their low CD14 expression, LPS-sensing, caspase-1 activation and P2X7R activity were comparable in non-classical monocytes to other subsets: their diminished ability to produce IL-1β instead arose from 50% increased IL-1β mRNA decay rates, mediated by Hsp27. These findings identify the Hsp27 pathway as a novel therapeutic target for the management of conditions featuring dysregulated IL-1β production, and represent an advancement in understanding of both physiological inflammatory responses and the pathogenesis of inflammatory diseases involving monocyte-derived IL-1β.

Monocytes are a heterogeneous population of immune cells accounting for approximately 10% of the blood mononuclear cell population. A key role of monocytes is to detect early signs of tissue damage or infection and respond by secreting pro-inflammatory cytokines to alert the immune system of the threat. One such cytokine is interleukin-1β (IL-1β). Production and secretion of IL-1β is essential in the response to bacterial, viral, fungal or parasitic infections, but dysregulated production of IL-1β contributes to the pathology of multiple diseases including atherosclerosis, type-2 diabetes, and various auto-immune diseases such as rheumatoid arthritis and colitis^1^. Thus the production of IL-1β must be tightly regulated within the cell types that produce it to support appropriate immunity and avoid immune-pathology.

The discovery of marked heterogeneity within the blood monocyte population adds another level of complexity to understanding IL-1β responses. Based on the expression of CD14 and CD16, human monocytes exist as classical (CD14++CD16−), intermediate (CD14++CD16+), and non-classical (CD14+CD16++) subsets^2^. Elevated numbers of monocytes, and the expansion of specific monocyte subsets, occurs in response to infection, but has also been observed in several chronic inflammatory diseases associated with increased IL-1β levels^3^. While detailed studies of monocyte subsets have revealed subset-specific cell surface markers and sets of differentially-expressed genes^4^,^5^,^6^,^7^, contradictory data exist on the capacities of the different subsets to produce cytokines, including IL-1β^3^,^4^,^7^ and the mechanistic basis for the observed differences are largely unknown.

Part of the difficulty in defining the roles of monocyte subsets in the production of IL-1β likely reflects the tight and complex regulatory mechanisms governing the cytokine’s generation and secretion. Similar to several inflammatory mediators, IL-1β is post-transcriptionally regulated: IL-1β mRNAs contain AU-rich elements (ARE) in their 3′ untranslated regions that render them susceptible to ARE-binding-protein-mediated degradation. The regulation of mRNA decay by ARE-binding proteins has been demonstrated in transgenic mouse models^8^, human monocytic^9^ and non-monocytic cell lines (including HeLa cells^10^ and the breast carcinoma cell line ZR-75-1^11^), and in primary human eosinophils^12^. Moreover, the ARE-binding proteins Tristetraprolin (TTP)^13^,^14^ and AU-rich element RNA-binding protein 1 (AUF1)^15^,^16^ are specifically involved in regulating inflammatory responses. AUF1, also known as heterogeneous nuclear ribonucleoprotein D (hnRNP D), binds to ARE-containing mRNAs and assembles together with translation initiation factors and heat shock proteins to recruit the mRNA degradation machinery and mediate mRNA decay^9^,^10^. A study done in human monocytic cell line THP-1 demonstrated the involvement of a complex formed by AUF1 with heat shock protein 27 (Hsp27) in the regulation of IL-1β^17^ though whether this pathway is also important during IL-1β production by monocytes is currently unknown.

Assuming that the IL-1β mRNA evades degradation, a second level of regulation of IL-1β production from immune cells exists during the conversion of immature pro-IL-1β into mature IL-1β. The “two-step secretion model” of IL-1β production states that a primary stimulus leads to increased synthesis and accumulation of pro-IL-1β and inflammasome components, and then a second stimulus induces caspase-1-mediated cleavage of pro-IL-1β to form mature IL-1β for secretion^18^. While macrophages seem to rely on the two-step model, monocytes may release mature IL-1β following a single stimulus: this was initially proposed to result from their endogenous ATP release, leading to constitutive caspase-1 activity^19^, but ATP-independent IL-1β secretion following TLR4 and TLR7/8 stimulation has since been documented^20^. The mechanisms underpinning ATP-independent IL-1β release in monocytes have not yet been entirely elucidated, but have been proposed to involve the TLR4 internalization-TRIF pathway^21^, TRIF-caspase-8^22^, and/or the cytoplasmic LPS-induced non-canonical caspase-4/5-dependent^23^ pathways. However, the studies by Piccini *et al*.^24^ and Ward *et al*.^20^, for example, used monocyte isolation methods leading to enrichment of specific subsets, and drew disparate conclusions on the involvement of endogenous ATP during IL-1β secretion: we therefore hypothesised that monocyte subsets might produce and release IL-1β via specialised, distinct, and largely-undefined mechanisms.

Here, we sought to identify whether primary human monocyte subsets differentially regulate IL-1β production, either at the level of mRNA stability and/or generation of mature IL-1β for secretion. Specifically we assessed the ATP-dependence of IL-1β secretion in monocyte subsets, and identified Hsp27-modulated ARE-mediated mRNA degradation as a key, subset-specific regulator of IL-1β production. Our findings suggest that the differential regulation of IL1-β production by human monocyte subsets may be a critical component of their distinct functional profiles in both health and disease.

## Results

### Non-classical monocytes release significantly less mature IL-1β upon LPS/LPS+BzATP stimulation

**Figure 1:**
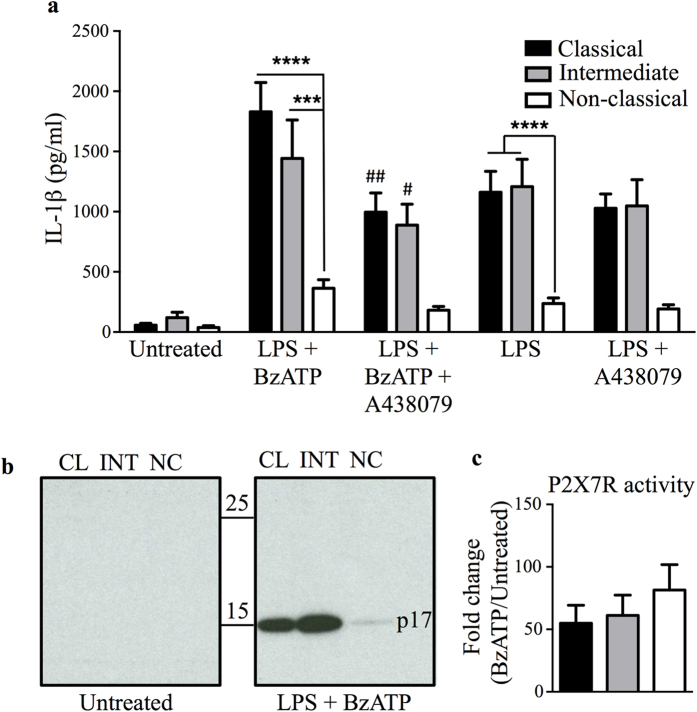
Non-classical monocytes secrete less mature IL-1β compared to other monocyte subsets following LPS and BzATP stimulation. (**a**) IL-1β ELISA on supernatants from monocyte subsets either untreated or stimulated with LPS for 24 h in the presence or absence of the P2X7 antagonist, A438079, followed by a secondary stimulation by BzATP in some conditions as indicated. Data plotted are mean ± SEM; n = 8. ****p < 0.0001 ***p < 0.001. ^##^*p* < 0.01 and ^#^*p* < 0.05 is comparison to the respective classical and intermediate subsets treated with LPS + BzATP. Statistics are calculated based on Two-way ANOVA with Tukey’s multiple comparison test (*p < 0.05). (**b**) Representative cropped immunoblot images for mature 17 kDa (p17) IL-1β in culture supernatants of monocyte subsets stimulated as described in (**a**). The samples were derived from the same experiment and were ran on the same gel. Full-length images are shown in Supplementary Fig. 3. (**c**) YoPro-1 dye uptake was measured by flow cytometry to reflect level of P2X7R activity, and is shown as the fold change of BzATP-induced YoPro-1 uptake normalized to the respective untreated subset uptake. Data plotted are mean ± SEM; n = 6 based on one-way ANOVA with Tukey’s multiple comparison test. CL: classical, INT: intermediate, NC: non-classical.

We first isolated primary human monocytes from the peripheral blood of healthy donors by fluorescence-activated cell sorting (FACS), and separated them into classical, intermediate, and non-classical subsets based on their relative expression of CD14 and CD16 (Supplementary Fig. 1). We then measured the amounts of IL-1β produced following a two-step stimulation protocol: initial priming by exposure to the bacterial cell wall component lipopolysaccharide (LPS) to induce the transcription and production of pro-IL-1βs; followed by treatment with BzATP, a synthetic substitute for extracellular ATP that acts via the P2X7 receptor (P2X7R) to stimulate the inflammasome complex/caspase-1 and alter membrane dynamics to promote the maturation and release of IL-1β^25^,^26^,^27^,^28^. LPS and BzATP treatment resulted in the production and release of IL-1β, with classical (1986.2 pg/ml ± 273.8) and intermediate (1510.7 pg/ml ± 313.8) monocytes secreting significantly more IL-1β than non-classical monocytes (681.9 pg/ml ± 243.1) (Fig. 1a). Immunoblot analysis also demonstrated a greater abundance of the bioactive 17 kDa form of IL-1β in the supernatants from classical and intermediate monocytes treated with LPS and BzATP compared to those from non-classical monocytes (Fig. 1b). Treatment with the P2X7R antagonist A438079 inhibited the BzATP-mediated IL-1β release step, and thus markedly decreased the amount of IL-1β in the supernatants of classical and intermediate monocytes treated with LPS and BzATP (Fig. 1a). The differential IL-1β secretion was observed not to be associated with a differential degree of cell death between the monocyte subsets (data not shown). To better understand the differences in production of IL-1β by the monocyte subsets, we measured the activity of P2X7R on their cell surfaces by flow cytometry (Fig. 1c). Upon stimulation by ATP or BzATP, P2X7R forms a pore which renders the cell selectively permeable to YoPro dye; we compared the pore forming activity of P2X7R on the three monocyte subsets by calculating the ratio of YoPro uptake in LPS + BzATP treated versus untreated cells (Fig. 1c), and observed no significant difference between subsets.

**Figure 2:**
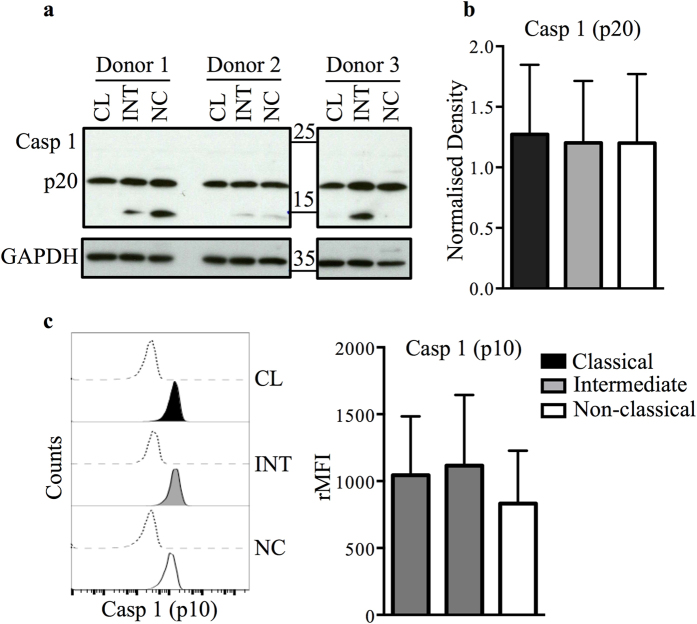
Caspase-1 activity is comparable between LPS-stimulated monocyte subsets. (**a**) The baseline activity of caspase-1 was assessed by immunoblotting of cleaved forms of caspase-1 (Casp 1, 20 kDa (p20), n = 3) on lysates of monocyte subsets from three different donors. The samples were derived from the same experiment and were ran on the same gel. Data shown are cropped images and the full-length images are in Supplementary Fig. 3. (**b**) Densitometric analysis of immunoblotting data (using an antibody specific to p20 caspase-1) of the 20 kDa band of caspase 1 (Casp1 (p20)) of LPS-treated monocyte subsets (n = 3). (**c**) Representative histogram plot showing intracellular cleaved 10 kDa caspase 1 (Casp 1 (p10)) expression either labelled with specific antibody (tinted) versus isotype-matched control (dashed line) in untreated monocyte subsets (left panel) and bar graph of the rMFI from 7 donors. Data plotted are mean ± SEM, one-way ANOVA with Tukey’s multiple comparison test. CL: classical, INT: intermediate, NC: non-classical, rMFI: relative mean fluorescence intensity.

As reported previously by Ward *et al*.^20^ in total monocytes, and suggested by the data in Fig. 1a, we also observed LPS-induced ATP-independent IL-1β secretion by monocyte subsets. Interestingly, the differential IL-1β release by all three monocyte subsets was also observed upon LPS treatment alone and was not altered even when the ATP pathway was inhibited with the addition of A438079 (Fig. 1a). Several previous studies have linked constitutive and non-canonical caspase-1 activation to ATP-independent IL-1β release^19^,^23^,^29^,^30^,^31^. We therefore measured constitutive and LPS-induced caspase-1 activation in monocyte subsets, first by immuno-blotting specifically for the active p20 caspase-1 subunit (Fig. 2a and b), then by flow cytometry to detect the active p10 caspase-1 subunit (Fig. 2c). Consistent with reports by other groups^19^,^32^, we detected constitutive caspase-1 activation in freshly isolated monocytes. However, we observed no significant difference in caspase-1 activation between monocyte subsets either immediately after isolation (Fig. 2a and c) or when treated with LPS for 24 hours (Fig. 2b). These results suggest that the attenuated IL-1β secretion in non-classical monocytes is not attributed to differential cleavage of pro-IL-1β to mature IL-1β by caspase-1, but is probably a consequence of an upstream regulatory mechanism.

### LPS-activated non-classical monocytes produce significantly less pro-IL-1β

**Figure 3:**
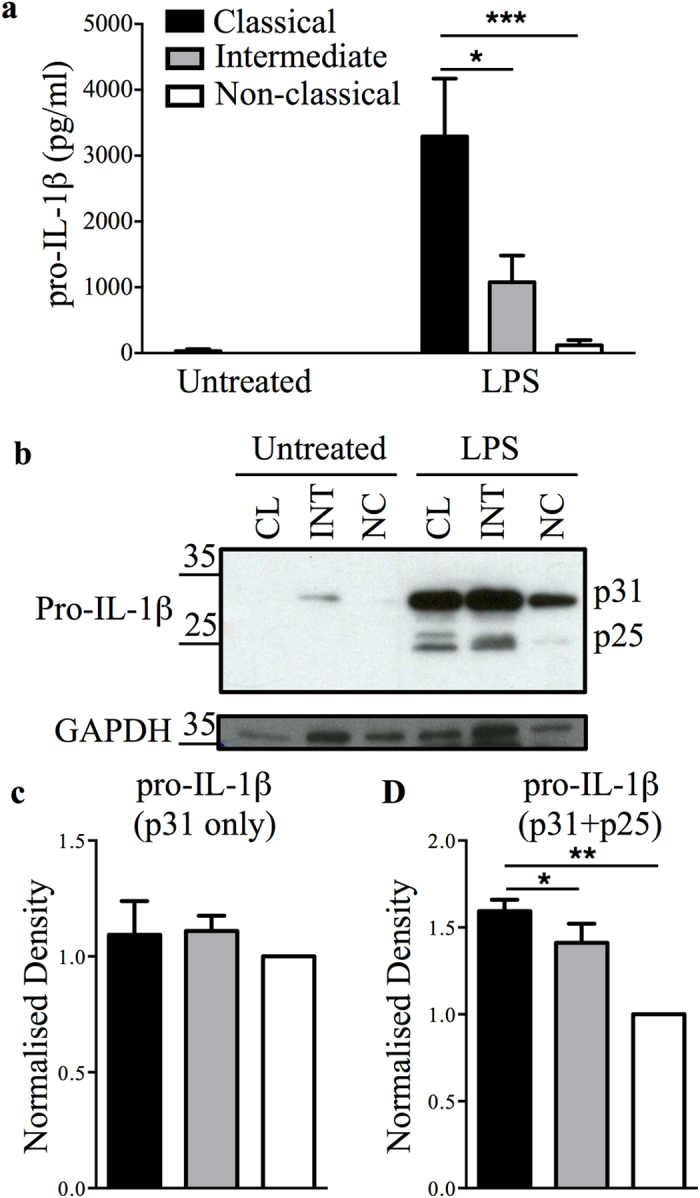
Non-classical monocytes produce less pro-IL-1β upon LPS stimulation. (**a**) Pro-IL-1β production measured by ELISA on supernatants of monocyte subsets either untreated or stimulated with LPS for 24 h. Data plotted are mean ± SEM; n = 3. *p < 0.05, ***p < 0.001 based on two-way ANOVA with Tukey’s multiple comparison test (**p < 0.01). (**b**) A representative cropped immunoblot image for pro-IL-1β expression in lysates of monocyte subsets treated as described in (**a**), with GAPDH as a loading control. The full-length image are shown in Supplementary Fig. 3. (**c**) Densitometric analysis of immunoblots for either 31 kDa (p31) alone or 31 kDa (p31) and 25 kDa (p25) together. Density was determined relative to the non-classical subset and normalized to GAPDH. Data plotted are mean ± SEM; n = 3. *p < 0.05, **p < 0.01 based on one-way ANOVA with Tukey’s multiple comparison test (**p < 0.01). CL: classical, INT: intermediate, NC: non-classical.

To determine whether the differential levels of IL-1β release by the monocyte subsets was due to differential synthesis of pro-IL-1β, we assessed the abundance of the precursor form of IL-1β (i.e. pro-IL-1β) in monocyte subsets by immuno-blotting and ELISA. Using an ELISA kit that predominately detects pro-IL-1β, we observed that non-classical monocytes accumulated significantly lower levels of pro-IL-1β upon LPS stimulation compared to the classical and intermediate subsets (Fig. 3a). Immuno-blotting consistently detected an additional unconventional precursor form of pro-IL-1β at around 25 kDa (p25), in addition to the conventional 31 kDa (p31) pro-IL-1β form (Fig. 3b). When densitometric quantification was performed (normalised to GAPDH) on the conventional p31 form alone, no significant difference in abundance was observed between the three subsets (Fig. 3c). However, when analysis was performed on both the p31 and p25 pro-IL-1β forms together, the non-classical subset showed significantly lower pro-IL-1β, which correlated with the relative levels of pro-IL-1β observed in the subsets by ELISA (Fig. 3d).

### Non-classical monocytes are not deficient in LPS sensing or induction of IL-1β mRNA

**Figure 4:**
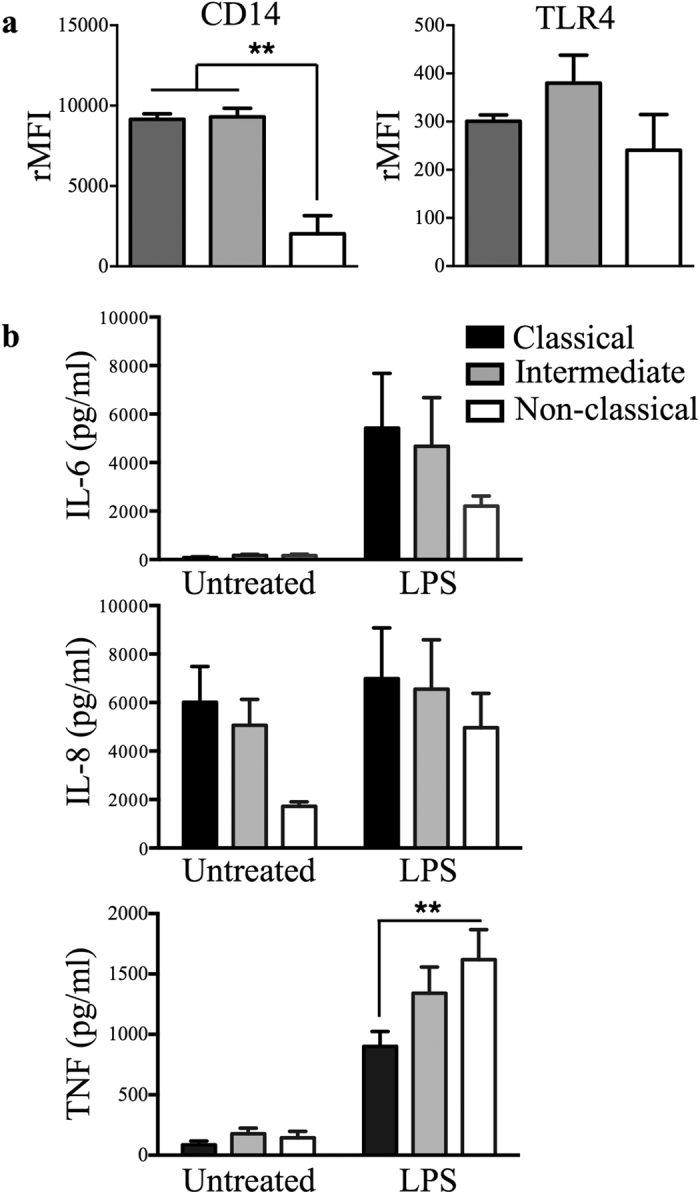
Low expression of CD14 does not affect the responsiveness of non-classical monocytes to LPS stimulation. (**a**) Flow cytometric analysis of cell surface expression of CD14 and TLR4 on monocyte subsets (n ≥ 2). (**b**) IL-6, IL-8 and TNF measured by ELISA on supernatants from monocyte subsets either untreated or stimulated with LPS for 24 h (n = 8). Data plotted are mean ± SEM, one-way (**a**) and two-way (**b**) ANOVA with Tukey’s multiple comparison test, **p < 0.01. rMFI: relative mean fluorescence intensity.

Since the production of pro-IL1β protein by the non-classical subset upon LPS stimulation is greatly reduced, we asked whether their ability to sense and respond to LPS was less than intermediate or classical monocyte subsets. LPS signals via the CD14/TLR4/MD2 receptor complex where CD14 facilities the transfer of LPS to the TLR4/MD2 complex^33^. Flow cytometric measurement of the intensity of surface CD14 expression revealed the expected pattern, with the non-classical subset showing significantly less CD14 expression compared to the classical and intermediate subsets, while TLR4 expression was comparable across subsets (Fig. 4a). To further characterise the responses of the three subsets to LPS, we measured the production of several LPS-induced pro-inflammatory cytokines, including IL-6, IL-8 and TNF, by ELISA (Fig. 4b). All subsets produced comparable amounts of IL-6 (Fig. 4b; top panel) and IL-8 (Fig. 4b; middle panel) following LPS exposure. However, the production of TNF was significantly higher in LPS-treated non-classical monocytes than classical monocytes, with the intermediate subset secreting an intermediate amount (Fig. 4b; bottom panel). These data suggest that the TLR4 pathway in non-classical monocytes is fully functioning and capable of transmitting stimulatory signals that are not significantly attenuated by the reduced levels of CD14 expression in these cells.

**Figure 5:**
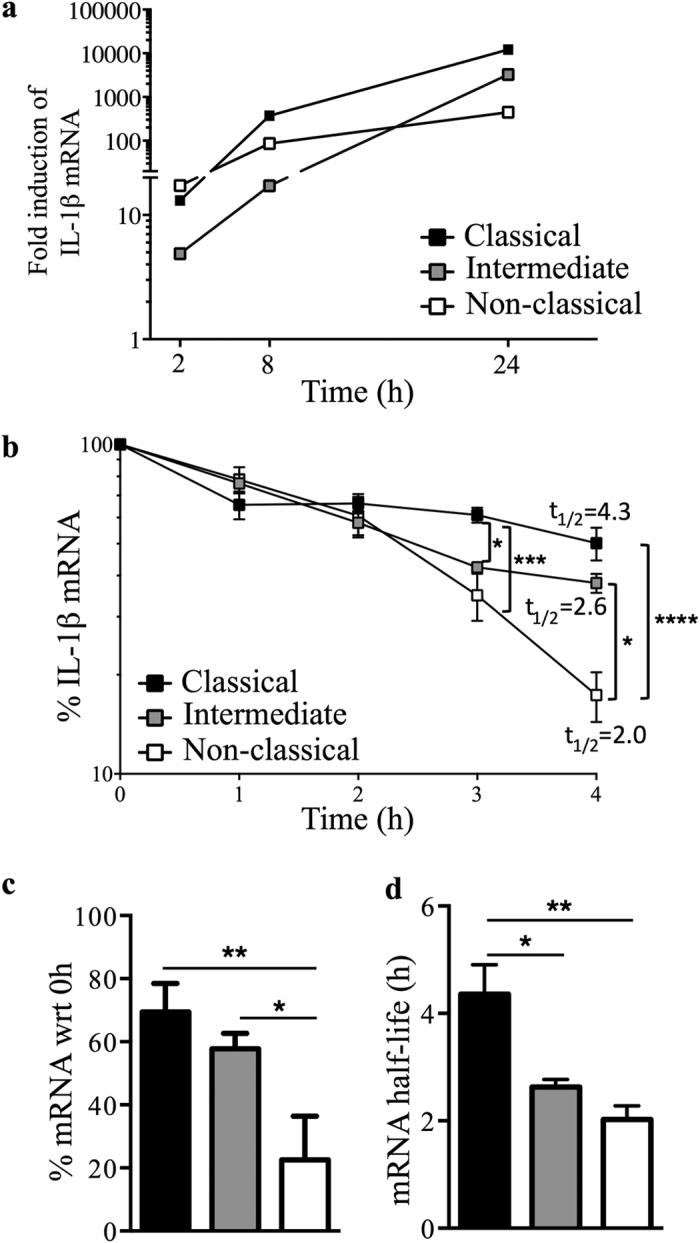
IL-1β mRNA degrades rapidly in non-classical monocytes. (**a**) Representative line graph showing the fold induction of IL-1β mRNA by monocyte subsets treated with 10 ng/ml LPS for the indicated times, (n = 3). (**b**–**d**) Subsets were stimulated with LPS for 2 h followed by addition of actinomycin D to stop *de novo* transcription. mRNA levels were determined by real-time qPCR at 1, 2, 3 and 4 h thereafter. Half-life (t_½_) of mRNA was calculated using non-linear regression, one phase decay analysis followed by two-way ANOVA statistical analysis. (**b**) The kinetics of mRNA decay and value shown is mean t_½_, (**c**) the percentage of remaining IL-1β mRNA at 4 h and (**d**) the half-life of IL-1β mRNA in monocyte subsets. Data plotted are mean ± SEM, n = 3, one-way and two-way ANOVA with Tukey’s multiple comparison test, *p < 0.05, **p < 0.01, ***p < 0.001 and ****p < 0.0001.

Having established that the TLR4 pathway in non-classical monocytes is fully functional, and given that our previous microarray study^4^ comparing the three monocyte subsets that were freshly isolated showed that the non-classical monocytes produced less IL-1β transcript, we next asked whether the lower levels of intracellular pro-IL-1β synthesis result from regulation at the point of transcription. When we measured the abundance of IL-1β mRNA in monocyte subsets exposed to LPS for 2 h and saw that the non-classical subset could markedly induced IL-1β expression to a higher level than classical and intermediate subsets (Fig. 5a and Supplementary Fig. 2), consistent with their observed responsiveness to LPS. However, despite the relatively lower response of classical and intermediate subsets at 2 h, they contained markedly more IL-1β mRNA than did non-classical monocytes by 24 h (Fig. 5a). These results demonstrate that *in vitro* exposure of primary human non-classical monocytes to LPS induces rapid activation that leads to early increases in IL-1β mRNA abundance; therefore the observed differences in production of IL-1β are unlikely to be explained by lower TLR4 receptor availability or attenuated signal transduction in these cells due to a lower CD14 expression.

### Monocyte subsets exhibit differential IL-1β mRNA stability following LPS exposure

Since the differential IL-1β protein levels in monocyte subsets are not the consequence of differential transcriptional control, we hypothesised that post-transcriptional regulatory mechanisms may be playing a part in the observed differences. As mRNA degradation is a post-transcriptional regulatory mechanism to control transcript copy number and thus protein levels, we measured the rate of mRNA decay following LPS stimulation of monocyte subsets. To determine the half-life of IL-1β mRNA in monocytes following 2 h of LPS stimulation, we inhibited the synthesis of new transcripts by the addition of actinomycin D and measured IL-1β mRNA levels at 1, 2, 3 and 4 h thereafter (Fig. 5b). At 4 h post-LPS treatment, non-classical monocytes showed a significantly lower percentage of remaining IL-1β mRNA than classical or intermediate monocytes (Fig. 5c). The LPS-induced IL-1β mRNA exhibited a half-life (t_½_) of 4.3 h, 2.6 h and 2 h in classical, intermediate and non-classical subsets, respectively (Fig. 5b and d). This equates to a 2.15-fold more stable transcript in classical monocytes and 1.3-fold in intermediate monocytes than non-classical monocytes: the IL-1β transcript degraded at a significantly faster rate in the non-classical subset, compared to classical and intermediate subsets (Fig. 5d).

### Hsp27 modulates IL-1β production in monocytes

**Figure 6:**
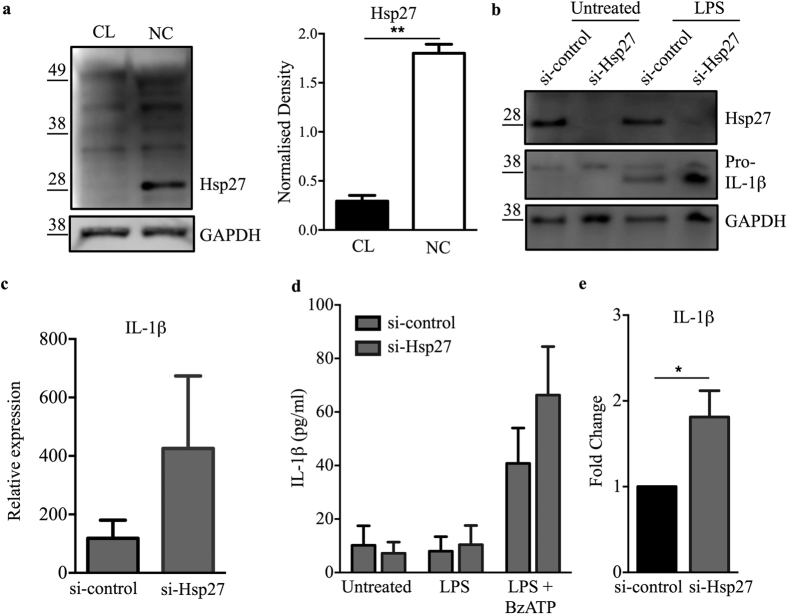
Hsp27 modulates IL-1β production in monocytes. (**a**) Immunoblot analysis of Hsp27 protein expression in classical (CL) and non-classical (NC) monocytes (left), and relative band densities from 2 combined experiments (right), with GAPDH as loading control. (**b**) Representative immunoblot of Hsp27 and pro-IL-1β (p31) in lysates from siRNA-transfected total monocytes for si-control (scrambled control siRNA) or si-Hsp27 (siRNA to Hsp27) either untreated or stimulated with LPS for 3 h. Data in (**a**) and (**b**) are cropped images and the corresponding full-length images are presentated in Supplementary Fig. 3. (**c**) Expression of IL-1β mRNA in siRNA-transfected total monocytes (n = 3). (**d**) Mature IL-1β release measured by ELISA of supernatants from siRNA-transfected total monocytes upon LPS stimulation in the presence or absence of BzATP (n = 4). (**e**) Fold change in IL-1β production by LPS and BzATP-treated total monocytes transfected with si-Hsp27, with respect to si-control. Data plotted are mean ± SEM, student *t*-test (**a** and **b**) and two-way ANOVA with Tukey’s multiple comparison test (**c**), *p < 0.05, **p < 0.01.

Next, we sought to understand the altered regulatory mechanisms underlying the differences in IL-1β mRNA stability between subsets. Prior studies identified Hsp27 as an essential subunit of the AUF1 protein complex, which regulates ARE-mediated mRNA decay of cytokines, including IL-1β, in monocytic cells such as THP-1^34^,^35^. We therefore assessed the protein levels of Hsp27 in freshly isolated monocyte subsets. Correlating with the higher IL-1β mRNA degradation rate, significantly more Hsp27 protein was detected in non-classical as compared to classical (1.8 ± 0.09 vs 0.29 ± 0.06) monocytes (Fig. 6a). To investigate the involvement of Hsp27 in the regulation of IL-1β gene expression and production, we performed siRNA-mediated knockdown of Hsp27 in the total monocyte population (since post-sorted monocyte subset numbers were insufficient and sorted cells were less amenable to the transfection procedure). The knockdown efficiency for Hsp27 was greater than 95% (Fig. 6b, top panel). We performed qPCR, immuno-blotting and ELISA to determine the effect of Hsp27 knockdown on IL-1β mRNA and protein levels and observed elevated IL-1β mRNA expression in Hsp27 knockdown monocytes compared to control siRNA transfected cells (Fig. 6c). Reduction in Hsp27 levels led to an increase of pro-IL-1β protein in LPS-treated monocytes by immuno-blotting (Fig. 6b). Pro-IL-1β was only observed in LPS-stimulated cells, demonstrating that the siRNA transfection itself did not activate the primary monocytes (Fig. 6b). When measured by ELISA, we detected ATP-mediated IL-1β release in LPS-stimulated monocytes, where Hsp27-knockdown cells released significantly more IL-1β (Fig. 6d and e). A previous study observed that, upon adherence in cell culture, primary monocytes gradually lost their ability to produce IL-1β in response to LPS and gained macrophage-like features, producing less IL-1 and exhibiting an increased requirement for ATP for that release^36^. In line with this, by ELISA we detected lower IL-1β levels and only ATP-mediated secretion in LPS-stimulated transfected monocytes. These results indicate that Hsp27 regulates IL-1β production in monocytes, and that the differential level of Hsp27 in monocyte subsets contributes to the differential subset-specific IL-1β production via mRNA decay.

## Discussion

IL-1β production by monocytes and macrophages is critical to initiate and maintain inflammatory responses, both in physiological immune responses and in pathological inflammation. The maturation and release of IL-1β has been extensively investigated for decades and it is now well established that the process of IL-1β secretion from monocytes and macrophages is substantially different: notably, monocytes are able to release IL-1β in response to TLR ligands alone^20^, while macrophages require a secondary stimulus^19^. A series of studies showed that ATP-independent caspase-1 activation and the consequent IL-1β secretion in monocytes occurs via non-canonical activation pathways involving the TRIF signalling pathway, cytoplasmic LPS-induced murine caspase-11 (caspase-4/5 in human) or caspase-8 mediated activation, resulting in enhanced IL-1β processing^21^,^22^,^29^,^30^,^31^. A recent report by Vigano *et al*.^23^ identified a caspase-5 mediated inflammasome pathway leading to caspase-1 activation as the regulatory mechanism of non-conventional LPS-induced IL-1 release from human primary monocytes. Monocyte heterogeneity is well recognised^37^ and most recent nomenclature divides them into 3 subsets: classical, intermediate and non-classical^2^. Several studies have provided insights into IL-1β production by human monocyte subsets but these reports have often been conflicting, potentially due to differences in gating strategies during subset isolation and in culture conditions^4^,^5^,^38^,^39^. The use of different stimulating factors (e.g. LPS from different species or strains) may also contribute to such discrepancies. Here we report that the classical and intermediate monocyte subsets release significantly higher levels of mature IL-1β upon LPS stimulation compared to the non-classical subset in both an ATP -dependent and -independent manner. Similar to the findings reported by Shantsila *et al*.^5^ and more recently by Edwan *et al*.^40^ and Sharma *et al*.^39^, non-classical monocytes treated with LPS produced the least IL-1β among the monocyte subsets. This is not due to an impaired response to LPS by the non-classical monocytes, as our results clearly demonstrate that they are active in producing other cytokines. To investigate the underlying regulatory mechanisms for differential IL-1β secretion between monocyte subsets, we performed a comprehensive mechanistic characterisation of IL-1β production.

The production of IL-1β is tightly regulated from the level of transcription through to secretion. First, we characterized the components of the canonical NLRP3 inflammasome-induced IL-1β cleavage and release in the three subsets: the ATP-induced inflammasome assembly, subsequent caspase-1 activation and caspase-1 mediated IL-1β maturation and release are all dependent on the ATP-gated cation channel, P2X7R. We measured P2X7R activity by the induction rate of BzATP-mediated dye uptake and found it to be similar in all three subsets. These data suggest that the downstream effects of inflammasome activation and caspase-1 cleavage may also be similar between the monocyte subsets. Previous reports showed that monocytes exhibit constitutive caspase-1 activity^19^, and similarly, we detected active caspase-1 using freshly-isolated and sorted monocytes, showing that levels of the active caspase-1 forms (p20 and p10) are comparable between subsets. The level of LPS-induced caspase-1 activation was also similar across the subsets. These observations led us to conclude that the subset-specific differences in IL-1β production were not a consequence of differences in inflammasome activation.

Upon investigating LPS-induced pro-IL-1β production by ELISA, we observed significantly less pro-IL-1β was produced by the non-classical subset. Intracellular pro-IL-1β levels, as assessed by immuno-blotting, were also lower in the non-classical monocytes. Using flow cytometry, Sharma *et al*.^39^ also reported a similar finding to ours. Interestingly, in addition to the precursor form of IL-1β (31 kDa), we detected an unconventional form (approx. 25 kDa; p25) in the cell lysates. Cleavage sites and cleavage products of pro-IL-1β are poorly characterised and documented in the literature. Until now, only an approx. 20 kDa (p20) form has been studied in macrophages^41^,^42^,^43^, microglial cells^44^,^45^,^46^ and endothelial cells^47^. According to Takenouchi *et al*.^45^, the p20 IL-1β form is generated extracellularly from leaked pro-IL-1β via cathepsin D cleavage under acidic conditions. It is believed that the p20 form competes with mature IL-1β for receptor binding to attenuate IL-1β signalling and reduce the adverse effects of inflammation. In a more recent publication, Edye *et al*.^46^ confirmed the findings of Takenouchi *et al*.^45^, showing the release of p20 from LPS primed THP-1 and mouse mixed glial cells at acidic pH (pH 6.2). Both studies demonstrated the involvement of cathepsin D by blocking the production of p20 with the cathepsin D inhibitor pepstatin A. Edye *et al*.^46^ reported additional bands (approximately 25 kDa) independently from pH and cathepsin D blocking but this p25 form was not investigated further. More recently Alfaidi *et al*.^47^ described a neutrophil elastase mediated, caspase-1 independent IL-1β cleavage mechanism, showing an increased expression of a p20 form upon neutrophil elastase treatment. Interestingly, the p25 form was differentially expressed in monocyte subsets, which correlated with subset differences in IL-1β production. Based on the microarray data previously published by our group^4^, the gene expression of neutrophil elastase is highest in the classical subset. In contrast, proteinase 3 and cathepsin G, which also process IL-1β^48^, were not altered between subsets. These findings suggest that neutrophil elastase may contribute to differential IL-1β processing in monocyte subsets. Functional characterisation of this novel IL-1β cleavage product and the involvement of neutrophil elastase are subjects of on-going studies in our group.

At both unstimulated and LPS stimulated conditions, the transcript level of IL-1β was significantly lower in the non-classical subset compared to the classical and intermediate subsets. Given the significantly different baseline levels of IL-1β mRNA in unstimulated monocyte subsets, we focused on IL-1β mRNA decay. Modulating mRNA stability is a rapid and efficient way of controlling gene expression and it has been reported as an important regulatory mechanism for cytokine expression (e.g. IL-6, IL-8, TNF or IL-1β) in immune cells^49^. We therefore characterised the degradation rate of IL-1β in monocyte subsets following LPS stimulation and observed the highest mRNA stability in the classical subset followed by the intermediate and non-classical monocytes. The half-life of IL-1β mRNA was significantly shorter in the non-classical subset and this resulted in a significantly less remaining IL-1β mRNA at 4 h following the inhibition of *de novo* transcription. Regulation of mRNA decay has been linked to protein complexes recognizing mainly AU-rich elements (ARE) in 3′UTR structures^50^,^51^, although GU-rich element mediated mRNA degradation has also been described^52^. In common with other inflammatory mediators, IL-1β mRNA was also shown to possess AU-rich structures, resulting in the rapid turnover of this transcript^53^. ARE-binding proteins (e.g. AUF1, HuR, TTP or KSRP) play a central role in the post-transcriptional regulation of ARE-containing mRNAs by affecting mRNA stability and degradation rate. AUF1 is an ARE-binding protein that mediates mRNA decay by interacting with translation initiation factors (eIF4G, PABP) and heat shock proteins (Hsp70 and Hsp27) to bind to ARE-containing mRNAs and to recruit the mRNA degradation machinery^10^,^34^,^35^. Knockout mouse studies^15^,^16^ reported the importance of AUF1 in the regulation of IL-1β; further studies on THP-1 pre-monocytic cells showed that Hsp27 is essential for AUF1-dependent mRNA decay^34^,^35^. Here we report that Hsp27 is differentially expressed between human primary monocyte subsets and that it regulates LPS induced IL-1β production in these cells by modulating mRNA decay. Knockdown of Hsp27 resulted in increased monocyte IL-1β secretion, suggesting that the higher Hsp27 expression and the subsequent Hsp27-mediated mRNA decay observed in the non-classical subset is the cause of the significantly lower IL-1β production.

In conclusion our study has identified the molecular basis for differential regulation of IL-1β in human monocyte subsets. We describe a novel control mechanism where Hsp27 is one of the main regulatory molecules of IL-1β mRNA stability, improving our understanding of the inflammatory response and offering potential therapeutic targets in diseases where IL-1β production becomes dysregulated and/or where monocyte subset proportions are altered.

## Methods

### Monocyte subsets isolation

Peripheral blood mononuclear cells (PBMCs) were isolated by Ficoll-Hypaque density centrifugation from apheresis cones, buffy coats or whole blood donated by healthy adult donors. Apheresis cones and buffy coats were provided by the Blood Donation Centre, Health Sciences Authority and National University Hospital Transfusion Centre, Singapore respectively. Ethical approvals for all blood sources and processes used in this study have been approved by either the National University of Singapore Institutional Review Board (Reference codes are NUS-IRB 08-352E, NUS-IRB 09-256, NUS-IRB 10-250) or the University of Sheffield Research Ethics Committee (UREC) under approval number SMBRER310. Subjects gave written informed consent in accordance to the Declaration of Helsinki. All experiments were carried out in accordance with the approved guidelines and regulations.

Monocyte isolation was carried out either by negative or positive magnetic selection depending on downstream applications. For total monocytes, they were positively isolated using CD14 microbeads (Miltenyi Biotec). For isolation of monocytes subsets, total monocytes were first enriched using non-monocyte depletion cocktail (CD15 and CD56 microbeads), CD3 microbeads and CD19 microbeads (Miltenyi Biotec). Separation of monocyte subsets was performed by fluorescence-activated cell sorting (FACS) using either BD^TM^ Influx, FACSAria II 4 lasers, FACSAria II 5 lasers or BD Jazz flow cytometers (BD Biosciences) based on the relative expression of CD14 and CD16 of NKp46-negative cells (Supplementary Fig. 1). Antibodies used for subset sorting were eFluor450 anti-human CD14 (61D3 clone, eBioscience), FITC anti-human CD16 (VEP13 clone, Miltenyi Biotec) and APC anti-human NKp46 (CD335, 9A2 clone, Miltenyi Biotec). Analysis of post-sort fractions showed that the purity of the separated subsets was consistently ≥98% for each subset.

### Primary cell culture and treatment

Primary blood monocytes were cultured in RPMI-1640 medium supplemented with 10% (v/v) heat inactivated, low endotoxin fetal calf serum (HI FCS-LE, Gibco), 1% (v/v) Penicillin-Streptomycin (P/S) and 1% (v/v) L-glutamine. Subsets of monocytes at 12,500 cells per well (50,000 cells/ml) were plated in 96 well plates and stimulated with 1 ng/ml LPS (purified from *E. coli*, serotype 0111:B4, TLRgrade, Enzo Life Sciences) for 24 h either in the presence or absence of 10 μM A438079 hydrochloride (P7X7R antagonist, Tocris Bioscience). 300 μM BzATP (P2X7R agonist, Sigma-Aldrich) was added for the final 20 minutes of incubation. Culture supernatants were collected and non-adherent cells removed by centrifugation. Samples were stored at −80 °C until analysis.

### Cytokine measurement by ELISA

A ‘sandwich’ based ELISA assay was used to determine the amount of treatment induced pro-IL-1β (Human pro-IL-1β/IL-1F2 Quantikine ELISA kit), mature-IL-1β (DY201), IL-6 (DY206), IL-8 (DY208) and TNF (DY210) production in monocyte subsets according to the manufacturer’s instructions (R&D Systems). The absorbance was detected at 450 nm using either a Tecan Infinite M200 or a Varioskan Flash Multimode (ThermoFiser Scientific) plate readers. Results were calculated by Prism 6.05 (GraphPad Software Inc.) using a four parametric logistic (4-PL) curve fit model.

### Gene silencing by siRNA

Small interfering RNA was delivered into total monocytes using Viromer technology according to the manufacturer’s protocol (Viromer Green, Lipocalyx). Monocytes were transfected with either control or Hsp27/HSPB1 siRNA (SMARTpool, ON-TARGETplus HSPB1 siRNA, Dharmacon) for 24 h. Knockdown of Hsp27 was confirmed by Immuno- blotting. For downstream analysis, transfected cells at 200,000 cells/ml were either left untreated, stimulated with 100 ng/ml LPS alone for 3 h or followed by 20 mins incubation with 300 μM BzATP. IL-1β production was measured by real-time qPCR, immuno-blotting and ELISA.

### Flow cytometry

Flow cytometry analyses were performed to characterise the cell surface expression of TLR4, CD14 and P2X7R and to determine intracellular levels of caspase-1 subunit p10. Briefly, 400,000 total monocytes were used for each labeling condition. For intracellular labeling, cells were fixed with 4% (v/v) PFA (20 mins, on ice) then permeabilized with 0.1% (v/v) triton X 100 - PBS (20 mins, room temperature). Labeling was performed in 1% (v/v) FCS-PBS for 15 mins at 4 °C in the dark. Antibodies used for standard cell surface/intracellular labeling were BV421 anti-human TLR4/CD284 (HTA125 clone, Biolegend), AF647 anti-human CD14 (HCD14 clone, Biolegend) and purified anti-human monoclonal P2X7R (kind gift from James Wiley, University of Melbourne, Australia^54^), purified anti-human caspase-1 (p10 subunit, ab62698 Abcam). Both P2X7R and caspase-1 antibodies were conjugated with fluorochrome using Lightning Link R-PE conjugation kit according to the manufacturer’s instructions (Innova Biosciences). Live/Dead fixable violet dye for live/dead staining was used according to the manufacturer’s instructions (Molecular Probes). Compensation was performed using anti-mouse Igk/negative control compensation particles set (BD Biosciences) and ArC Amine reactive compensation bead kit (Molecular Probes), according to the manufacturer’s instructions.

### P2X7R activity

P2X7R activity as assessed by pore dilation leading to permeability of the receptor to large fluorescent dyes like YoPro was determined. 400,000 total monocytes were washed with HEPES-buffered saline (130 mM NaCl, 5 mM KCl, 20 mM HEPES (pH 7.4), 10 mM D-glucose and 0.1% (w/v) BSA) and centrifuged at 450× *g* for 5 mins. Surface staining was performed in HEPES buffered saline for 15 mins at room temperature. Cells were then washed twice with potassium glutamate buffer (130 mM potassium glutamate, 5 mM KCl, 20 mM HEPES (pH 7.4), 10 mM D-glucose and 0.1% (w/v) BSA). To measure the receptor activity, the cell pellet was resuspended in 300 μl potassium glutamate buffer containing 0.25 μM YoPro-1 Iodide (Life Technologies) with or without 100 μM BzATP and 10 μM A438079 hydrochloride and incubated for 20 mins at 37 °C in the dark. An unstained (no surface staining and no Yo-Pro) control was prepared by resuspending cells in 300 μl potassium glutamate buffer. The reaction was stopped by adding 100 μl ice-cold HEPES buffered saline containing 20 mM MgCl_2_ and then washed with HEPES-buffered saline. Live/Dead staining was performed and cells were then resuspended in FACS buffer (1x PBS and 5% (v/v) FCS) for flow cytometry analysis.

### Immuno-blotting

Monocyte subsets at 62,500–125,000 cells per well in 250 μl were placed into 96-well plates. After treatment, the plate was centrifuged (800× *g*, 5 mins) to pellet any non-adherent cells. Supernatants were discarded while cell pellets were lysed in 25–50 μl 1x RIPA (Radio Immuno Precipitation Assay, Sigma) buffer supplemented with protease inhibitors (Complete mini EDTA free Protease Inhibitor cocktail tablets, Roche). Samples were collected and stored at −80 °C until analysis. Cell lysates were sonicated and incubated on ice for 20 mins with intermittent vortexing then centrifuged at 12,000× *g* for 8 mins at 4 °C; the supernatant was transferred to a clean tube. Supernatants were filtered through 50 kDa filtration columns (Vivaspin column, Sartorius Stedium Biotech) by spinning at 14,000× *g* for 8 mins followed by concentrating the flow-through using 10 kDa filtration columns (Vivaspin column) by spinning at 12,000× *g* for 3 mins before 4x Laemmli loading buffer (BioRad) was added. Samples were denaturation at 95 °C for 5 mins followed by incubation on ice for 10 mins before separating on a 15% SDS-PAGE gel and transferred onto PVDF membrane (Amersham, Hybond-P). Membranes were rinsed with PBS-T (1x PBS and 0.1% (v/v) Tween-20) then blocked with 5% (w/v) skimmed milk in PBS-T for 1 h at room temperature with gentle shaking. After blocking, the membrane was rinsed with PBS-T then incubated with antibodies in 5% (w/v) milk-PBS-T at 4 °C overnight. Primary antibodies used were mouse anti-human IL-1β (MAB201, R&D Systems), rabbit anti-human caspase-1 (p20 subunit, ab17820 Abcam), mouse anti-human Hsp27 (G3.1, ab2790, Abcam) and mouse anti-human GAPDH (clone 0411, Santa Cruz). The primary antibodies were detected using HRP conjugated goat anti-mouse/rabbit IgG antibody (Dako). For Hsp27, insufficient intermediate monocyte numbers were obtained post-cell sorting for assessment. Densitometry analysis was performed using ImageJ software; data were normalised to GAPDH.

### Real-time qPCR and the determination of mRNA half-life

200,000–350,000 cells/ml of sorted monocyte subsets were treated with 10 ng/ml LPS for 2 h in complete medium. For gene expression measurements, cells were collected after 2 h of treatment. For mRNA degradation rate determination, cells were treated with 10 μg/ml actinomycin D (Sigma Aldrich) for a further 1, 2, 3 and 4 h post-LPS treatment to arrest further *de novo* RNA synthesis before collection. Treated cells were pelleted at 900× *g* for 5 mins, supernatant was removed and cell pellets resuspended in 700 μl Qiazol (Qiagen). Samples were stored at −80 °C then RNA was extracted using a Qiagen RNeasy Micro kit (Qiagen), according to the manufacturer’s instructions. RNA concentration and quality were measured by Nanodrop 1000 Spectrophotometer (Thermo Scientific 300 ng RNA per sample was reversed transcribed to obtain cDNA using iScript^TM^ Reverse Transcription Supermix (Bio-Rad) according to the manufacturer’s instructions. Real-time qPCR was performed using KAPA SYBR FAST qPCR Universal Master mix (KAPA Biosystems) with the following validated primer pairs:

IL1B-FW 5′AACCTATCTTCTTCGACACATGGGATA3′

IL-1B-RV 5′CAAGGCCACAGGTATTTTGTCATTACT3′

HPRT-FW 5′CAAGGGCATATCCTACAACAAAC3′

HPRT-RV 5′CTTTGCTTTCCTTGGTCAGG3′

The IL-1β expression level in samples from Hsp27 knockdown experiments was determined using KAPA PROBE FAST qPCR Universal Master mix (KAPA Biosystems) using the following primers with TaqMan probes:

IL1B-FW 5′ACAGATGAAGTGCTCCTTCCA3′

IL1B-RV 5′GTCGGAGATTCGTAGCTGGAT3′

Probe: 5′ FAM CTCTGCCCTCTGGATGGCGG TAMRA 3′

GAPDH-FW 5′GCCTTCCGTGTCCCCACT3′

GAPDH-RV 5′TGAGGGGGCCCTCCGACG3′

Probe: 5′ FAM CCTGCTTCACCACCTTCTT

A negative control, using dH_2_O in place of cDNA samples, was set up in each run. Amplification was conducted using an ABI7900HT Fast (Applied Biosystems) or Bio-Rad CFX (Bio-Rad) Real-Time qPCR Systems. The ∆∆Ct method was used for analysis. The mRNA degradation rate was calculated using non-linear regression, one phase decay model.

### Statistical analysis

All data were analysed using Prism 6.05 (GraphPad Software Inc.). Non-linear regression analysis was performed using the one-phase decay exponential equation. Statistical significance was determined by one-way or two-way ANOVA with Tukey’s multiple comparison post-test or student *t*-test (two-tailed). ‘N’ represents the number of distinct donors in each experiment.

## Additional Information

**How to cite this article**: Hadadi, E. *et al*. Differential IL-1β secretion by monocyte subsets is regulated by Hsp27 through modulating mRNA stability. *Sci. Rep.* **6**, 39035; doi: 10.1038/srep39035 (2016).

**Publisher's note:** Springer Nature remains neutral with regard to jurisdictional claims in published maps and institutional affiliations.

## Supplementary Material

Supplementary Information
