# Supplementary material for: Differential IL-1β secretion by monocyte subsets is regulated by Hsp27 through modulating mRNA stability
Source: Sci Rep. 2016 Dec 15;6:39035. doi: 10.1038/srep39035 (PMC5157043; doi:10.1038/srep39035)
Supplement: Supplementary Information [file srep39035-s1.pdf]

Differential IL-1β secretion by monocyte subsets is regulated by Hsp27 through modulating mRNA stability.

Eva Hadadi[1,2], Biyan Zhang[2], Kajus Baidžajevas[1], Nurhashikin Yusof[2], Kia Joo Puan[2], Siew Min Ong[2], Wei Hseun Yeap[2], Olaf Rotzschke[2], Endre Kiss-Toth[1#], Heather Wilson[1#*], Siew Cheng Wong[2#*]

[1]University of Sheffield, Dept of Infection, Immunity & Cardiovascular Disease (IICD), Sheffield, UK. [2]Singapore Immunology Network (SIgN), Agency for Science, Technology and Research (ASTAR), Singapore. [#]Equal Contributions

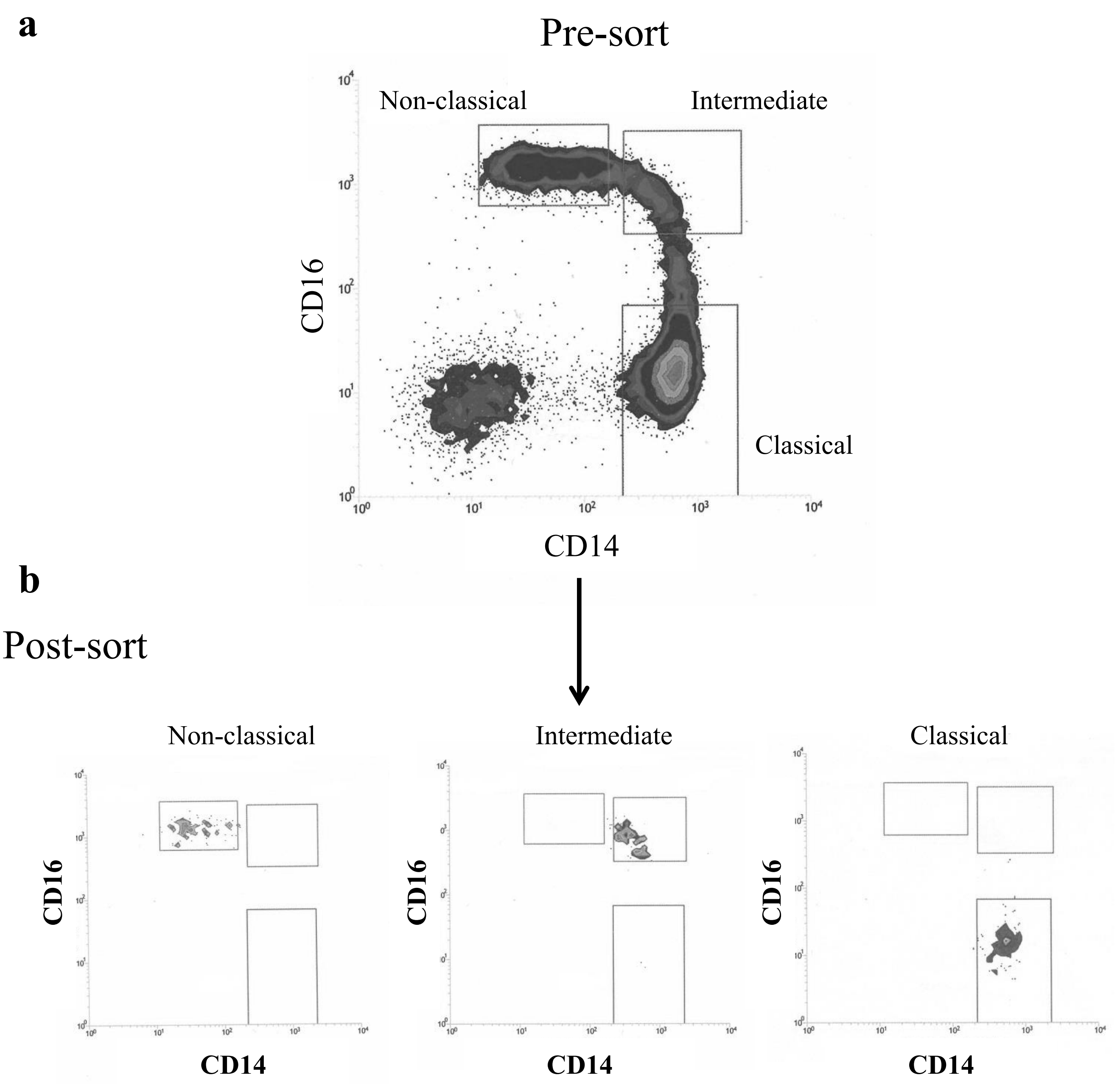

a
Pre-sort
Non-classical
Intermediate
Classical
CD16
CD14
b
Post-sort
Non-classical
Intermediate
Classical
CD16
CD14
CD16
CD14
CD16
CD14

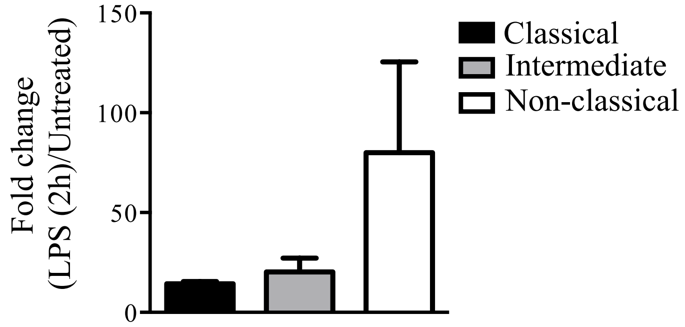

Fold change
(LPS (2h)/Untreated)
150
100
50
0
Classical
Intermediate
Non-classical

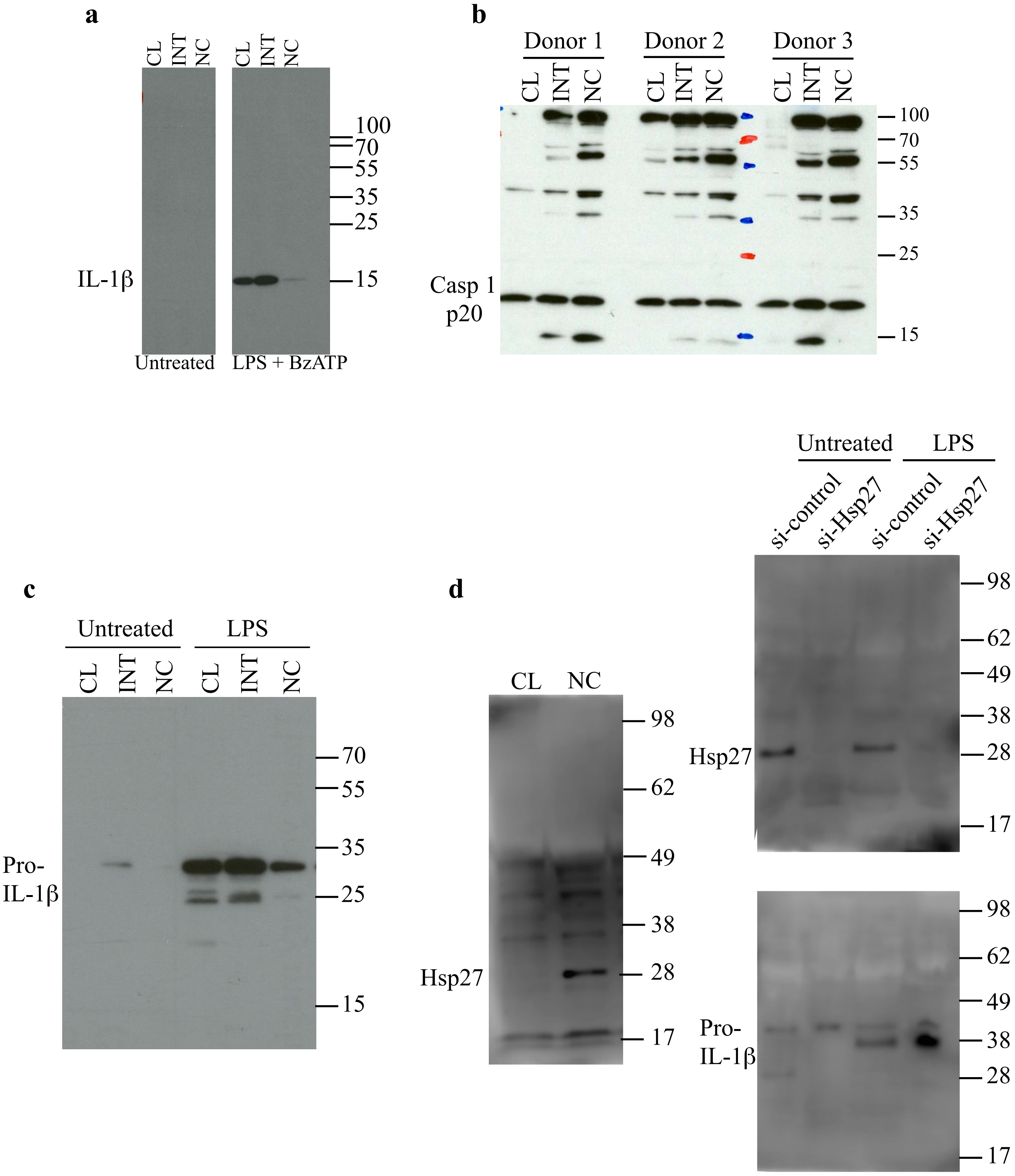
a
CL INT NC CL INT NC
100
70
55
35
25
IL-1β
15
Untreated
LPS + BzATP
b
Donor 1
Donor 2
Donor 3
CL INT NC CL INT NC CL INT NC
100
70
55
35
25
Casp 1
p20
15
c
Untreated
LPS
CL INT NC CL INT NC
70
55
35
Pro-
IL-1β
25
15
d
CL NC
98
62
49
38
Hsp27
28
17
Untreated
LPS
si-control si-Hsp27 si-control si-Hsp27
98
62
49
38
Hsp27
28
17
98
62
49
Pro-
IL-1β
38
28
17

Figure Legend

Supplementary Figure 1: Gating Strategy for monocyte subsets isolation

(a) Representative density plots of the three human monocyte subsets gated based on their relative expression of CD14 and CD16. (b) Post-sort runs of separated subsets showing purity over 98%.

Supplementary Figure 2: Fold induction of IL-1β mRNA in monocyte subsets following 2h LPS stimulation

Subsets were stimulated with 10 ng/ml LPS for 2h and the induction of gene expression was determined by real-time qPCR. Data plotted are mean ± SEM, n = 3, one-way ANOVA with Tukey's multiple comparison test.

Supplementary Figure 3: Full-length Western blot data

The figure shows full-length Western blot images for (a) IL1β, (b) caspase1 p20, (c) Pro-IL1β; (d) Hsp27 and pro-IL1β.
